# Blue cheese-making has shaped the population genetic structure of the mould *Penicillium roqueforti*

**DOI:** 10.1371/journal.pone.0171387

**Published:** 2017-03-01

**Authors:** Jeanne Ropars, Manuela López-Villavicencio, Alodie Snirc, Sandrine Lacoste, Tatiana Giraud

**Affiliations:** 1 Institut de Systématique, Évolution, Biodiversité, UMR 7205 CNRS, MNHN, UPMC, EPHE, Muséum National d’Histoire Naturelle, Paris, France; 2 Ecologie Systématique Evolution, Université Paris-Sud, CNRS, AgroParisTech, Université Paris-Saclay, Orsay, France; University of Torino, ITALY

## Abstract

**Background:**

*Penicillium roqueforti* is a filamentous fungus used for making blue cheeses worldwide. It also occurs as a food spoiler and in silage and wood. Previous studies have revealed a strong population genetic structure, with specific traits associated with the different populations. Here, we used a large strain collection from worldwide cheeses published recently to investigate the genetic structure of *P*. *roqueforti*.

**Principal findings:**

We found a genetic population structure in *P*. *roqueforti* that was consistent with previous studies, with two main genetic clusters (W+C+ and W-C-, i.e., with and without horizontal gene transferred regions *CheesyTer* and *Wallaby*). In addition, we detected a finer genetic subdivision that corresponded to the environment and to protected designation of origin (PDO), namely the Roquefort PDO. We indeed found evidence for eight genetic clusters, one of the cluster including only strains from other environments than cheeses, and another cluster encompassing only strains from the Roquefort PDO. The W-C- and W+C+ cheese clusters were not the most closely related ones, suggesting that there may have been two independent domestication events of *P*. *roqueforti* for making blue cheeses.

**Significance:**

The additional population structure revealed here may be relevant for cheese-makers and for understanding the history of domestication in *P*. *roqueforti*.

## Introduction

Cheese making by early Neolithic farmers was a major advance in food processing, allowing to preserve milk in a non-perishable, transportable form, and making milk more digestible for adults as cheese contains much less lactose than fresh milk [1]. Earliest cheese making footprints date from the sixth millennium BC in Poland, with findings of milk fat’s presence in sieve vessels [2], and from the early Bronze Age (ca. 3800 years old), with the discovery of residues of old cheese in tombs [3].

For making the variety of blue cheeses worldwide, such as the very famous French Roquefort, English Stilton, Spanish Cabrales, Danish Danablue or Italian Gorgonzola, industrials use specific strains of the fungal ascomycete species, *Penicillium roqueforti*. Originally, *P*. *roqueforti* was not inoculated during blue cheese production but contaminated the milk spontaneously with spores from the environment. Since the end of the 18th century, *P*. *roqueforti* asexual spores (conidia) are inoculated into the cheese curd [4,5] at the beginning of the cheese-making process. Spores to be inoculated were initially collected from naturally-rotten bread, thus coming from the environment, likely from wild, sexually-recombining populations of the fungus in caves or farms [4,5]. Then, inocula that gave good cheeses were selected and inoculated into the breads for clonally multiplying the spores. Since these last 40 years, to prevent sanity issues and to make the cheese maturation process more replicable and reliable, the inoculated strains are cultivated *in vitro* from monospore isolations [4,5]. This represents recent strong selection of a few clonal lineages and subsequent exclusive asexual culturing.

*Penicillium roqueforti* also occurs as a common spoilage agent in food (e.g., bread, fruits), and even in refrigerated stored food due to its capacity to tolerate cold temperatures, low oxygen concentrations, alkali and weak acid preservatives. *Penicillium roqueforti* is also found in other environments such as silage or occasionally in wood, but its natural ecological niche is still unknown [6–8]. Previous population studies have shown the existence of differentiated genetic clusters within *P*. *roqueforti* [9–11], revealing the existence of two main genetic clusters, each subdivided into three sub-populations [10]. The first main cluster contained exclusively strains isolated from cheese that carried two horizontally-transferred regions *Wallaby* (W) and *CheesyTer* (C), shared with several other *Penicillium* species isolated from cheese environment, such as *P*. *camemberti*, the fungus used to make Brie and Camembert cheeses [12,13]. The *Wallaby* and *CheesyTer* genomic islands seemed to encompass crucial metabolic genes providing competitive advantage and a better use of the cheese substrate, as revealed by experiments showing that strains carrying *Wallaby* and *CheesyTer* (i.e., W+C+) had a higher growth rate on cheese medium than strains without them (i.e., W-C-), and a lower growth rate on poor medium [13]. Strains belonging to the second main cluster were isolated from diverse environments, *i*.*e*. cheese, but also wood, silage, and none of them carried either *Wallaby* or *CheesyTer*.

More recently, population genetics analyses on a larger collection of strains showed that this second cluster of W-C- strains showed further genetic subdivision, separating strains collected in cheese from those collected in other environments [11]; these two clusters were thus renamed the W-C- cheese and the W-C- non-cheese clusters, respectively. In terms of genetic diversity, the W+C+ cheese cluster showed much lower diversity than the W-C- non-cheese cluster, the W-C- cheese cluster being intermediate [11]. The different cheese populations seemed to correspond to blue cheese types and to be morphologically different in terms of color and growth rates, suggesting that the population structure has been shaped by different cheese-making processes or that different populations were selected by producers for making the diverse cheese types [9]. The different *P*. *roqueforti* genetic clusters still belong to a single species, as supported by the genealogical concordance phylogenetic species recognition (GCPSR) criterion [9] and by interfertility between clusters [11]. However, the two cheese clusters displayed degeneration in terms of fertility, with the W-C- cheese cluster showing mostly pre-mating sterility and the W+C+ cheese cluster mostly post-mating sterility [11].

Identifying a finer genetic subdivision within *P*. *roqueforti*, with possibly different genetic clusters of cheese-making strains used for different types of cheeses, harboring specific morphologic or metabolic traits, would be both of high applied interest and of fundamental importance, for understanding the process of domestication. Here we aimed at investigating whether a finer genetic subdivision could be detected within *P*. *roqueforti*, and in particular according to the type of cheeses. To this goal, we used the recently published large collection of 240 *P*. *roqueforti* strains isolated from worldwide cheeses and from other environments [11] and looked for the finest genetic structure in the dataset. In the previous study that published the strain collection and the genetic dataset [11], a goal was to assess the degree of fertility between the three main genetic clusters, as well as the fertility of cheese strains; therefore, only the strongest genetic subdivision was displayed. Here, we looked for the finest genetic subdivision in the dataset.

## Material and methods

### Strain collection and genetic data

We used the set of 240 *P*. *roqueforti* strains previously analysed [11], that have been deposited in the public French LCP (Laboratoire de Cryptogamie, Paris) collection at the National Museum of Natural History, where all the strains analysed here are permanently and publicly available. Among the 240 strains, 210 strains were isolated from near a hundred of different blue cheeses collected worldwide (*e*.*g*., Roquefort, Gorgonzola, Stilton, Cabrales, Blue Gouda, Danish blue, Cheddar blue), 28 strains were isolated from other environments (*e*.*g*., wood, silage, rotten fruits) and two isolates were of unknown origin [11].

All the 240 strains had been genotyped previously [11] using the eight polymorphic microsatellite markers giving the clearest patterns among those described [10]: Proq12, Proq13, Proq73, Proq74, Proq78, Proq80, Proq81 and Proq88. Genomic DNA had been extracted from fresh mycelium of the single-genotype strains using the Qiagen DNeasy Plant Mini Kit (Qiagen, Ltd. Crawley, UK). Microsatellite markers were amplified by multiplex PCR, with the Multiplex PCR Kit (Qiagen). Electrophoresis genotyping by capillary fractionation was carried out at INRA Clermont-Ferrand (Plateforme Strategique INRA, Ibisa 2009, ISO9001:2008). The profiles had been analysed with GENEMAPPER Software Version 4.0 (Applied Biosystem, Ville bon-sur-Yvette, France). The collection was also screened for the presence/absence (noted +/-) of the two horizontally-transferred genomic islands [11] that have been suggested to be involved in adaptation to cheese environment for the industrial strains [12,13], *Wallaby* (W) and *CheesyTer* (C).

### Genetic data and population genetics analyses

Individual-based Bayesian clustering method implemented in STRUCTURE 2.3.3 [14] was used to assign strains to the different genetic clusters. Ten independent analyses were carried out for each number of clusters, from *K* = 1 to *K* = 10, using admixture models and 500 000 MCMC iterations, after a burn in of 50 000 steps. The output was processed using CLUMPP v1.1.2 [15], to identify clustering solutions in replicated runs for each value of K. Population structure was then displayed graphically with DISTRUCT v1.1 [16]. We computed the *deltaK* statistics [17] via the Structure Harvester website [18] (http://taylor0.biology.ucla.edu/structureHarvester/), to identify the *K* value corresponding to the strongest structure. A discriminant analysis of principal components (DAPC) was also computed using the *adegenet* package [19] implemented in the R software [20], for the two most relevant K values for our dataset, *i*.*e*., *K = 3* and *K = 8*. *F*_*ST*_ and Fisher’s exact tests of population differentiation were computed using Genepop on the web [21,22]. The strain network was inferred from the distance matrix obtained from the microsatellite dataset by using the Neighbor-Net method in the Splitstree software (http://www.splitstree.org/).

## Results

**Fig 1:**
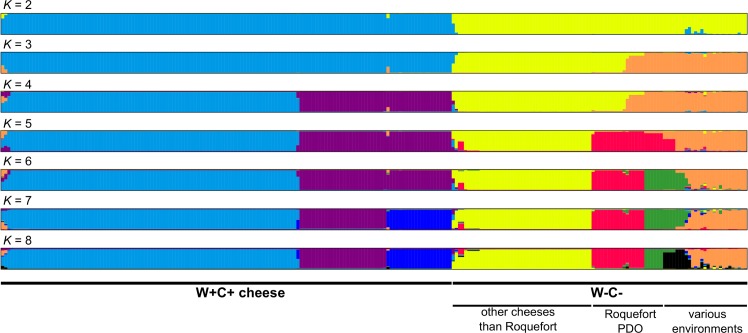
Population structure of *Penicillium roqueforti*. Coefficients of membership in various gene pools inferred with the STRUCTURE program of our 240 *Penicillium roqueforti* strains, based on the eight polymorphic microsatellite markers. STRUCTURE was run from K = 1 to K = 10 (showed up to K = 8 as no additional well-delimited cluster was revealed above). For K = 7 and K = 8 only the main mode are shown, where simulations could find the required number of clusters. The legend below the barplots indicates whether strains carry the *Wally* and *CheesyTer* genomic islands in their genomes (W+C+) or not (W-C-) and whether they have been collected from cheeses, belonging or not to the Roquefort protected designation of origin (PDO), or from other environments, such as silage or spoiled food.

**Fig 2:**
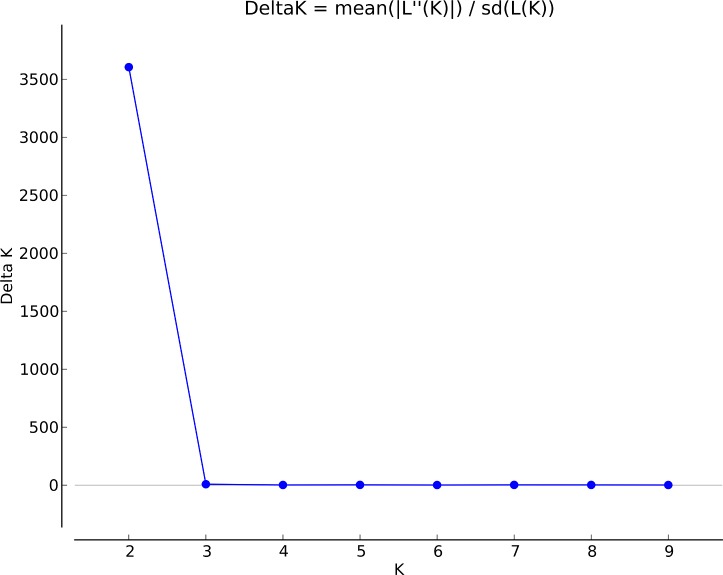
Implementation of the Evanno’s method for detecting the number of K groups that best fit the data. According to the **Δ**K, K = 2 represents the strongest structure in our dataset.

We reinvestigated available genetic data of 240 strains of *P*. *roqueforti* isolated from cheeses (*N* = 210) but also from other environments such as silage or wood (*N* = 28). The population structure inferred showed genetic subdivision with well-delimited clusters up to *K* = 8 (Fig 1). The *delta K* value pointed to *K* = 2 as the strongest structure level in the data set (Fig 2), separating strains exclusively isolated from dairy environments, carrying both horizontally-transferred regions *Wallaby* and *CheesyTer* (i.e., the W+C+ strains), from strains lacking both (i.e., the W-C- strains). At *K* = 3, the W-C- strains were split into two well-delimited clusters, one with only cheese strains and a second with strains from various environments (Fig 1), as shown previously [11]. At *K* = 4, the W+C+ cheese cluster was subdivided into two well-delimited clusters. At *K* = 5, strains included in the “various environments” cluster (revealed at *K* = 3) were further split into two clusters, one including mainly strains isolated from cheeses belonging to the Roquefort protected designation of origin (PDO), and the other one including mainly non-cheese strains (*e*.*g*. strains from silage, wood, food spoilers). At *K* = 6, this non-cheese cluster split into two clusters without obvious segregation according to environment of collection. At *K* = 7, one of the two W+C+ cheese cluster split into two clusters, one including mainly strains isolated from Gorgonzola cheeses. Finally, at *K* = 8, strains isolated from the Roquefort PDO were further split. From K = 9 and above, no further well-delimited cluster could be identified.

*K* = 8 thus represents the finest genuine genetic structure detectable in our dataset (Fig 1), and it identifies two genetic clusters of W-C- strains from various substrates (the orange cluster including only strains from non-cheese environments, i.e. isolated from silage, contaminated food and wood and the black one including mainly strains from the Roquefort PDO, but a few contaminant strains), three W-C- cheese clusters (two of them, i.e. the red and green clusters, including virtually only strains from the Roquefort PDO and the yellow cluster including a few W+C+ strains) and three W+C+ cheese clusters (with no obvious segregation according to geographical origin or type of cheese). S1 Table gives the assignment with precise information on the strains.

The *F*_*ST*_ values between the eight clusters showed that they represented strongly differentiated clusters (Table 1). Fisher’s exact tests further supported the differentiation in eight populations, indicating highly significant gene differentiation between all pairs of populations (P<10^−7^).

**Table 1:** Mean *F*_*ST*_ values across microsatellite markers between the eight genetic clusters in *Penicillium roqueforti*. The names of the cluster indicates whether they possess the *Wally* and *CheesyTer* genomic islands (W+C+) or not (W-C-) and whether they have been collected from cheeses, belonging or not to the Roquefort protected designation of origin (PDO), or from other environments, such as silage or spoiled food.

		W+C+ cheese			W-C-Various environments			
		1	2	3	4	5	6	7
W+C+ cheese	2	0.2						
	3	0.3	0.3					
W-C-Various environments	4	0.7	0.7	0.6				
	5	0.7	0.8	0.6	0.4			
	6	0.7	0.7	0.5	0.3	0.3		
	7	0.8	0.7	0.5	0.3	0.4	0.3	
W-C- cheese	8	0.8	0.8	0.7	0.3	0.6	0.6	0.5

**Fig 3:**
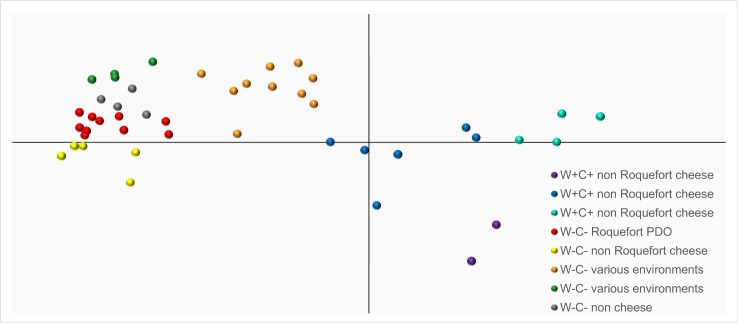
Discriminant analyses of principal components (DAPC) of *Penicillium roqueforti* strains at K = 8. The colors correspond to those in Fig 1 of the clusters identified using Structure. The name of the clusters indicate whether they possess the *Wally* and *CheesyTer* genomic islands (W+C+) or not (W-C-) and whether they have been collected from cheeses, belonging or not to the Roquefort protected designation of origin (PDO), or from other environments, such as silage or spoiled food.

The discriminant analysis of principal component (DAPC), assuming no particular model such as panmixia, also discriminated eight genetic clusters that mostly corresponded to the clusters found with the Bayesian clustering method implemented in STRUCTURE (Fig 3). The three W+C+ cheese clusters were grouped close one to each other. The W+C+ and W-C- cheese clusters appeared at opposite edges of the first axis, indicating that they were not the most closely related ones, suggesting that there may have been two independent domestication events of *P*. *roqueforti* for making blue cheeses. The orange cluster, including W-C- strains isolated from silage, wood, food spoiler and even cheese, appeared closest to the W+C+ cheese strains along this axis, while the W-C- cheese strains were farther left. The green and grey clusters including W-C- strains from mostly silage or spoiled food, were grouped close one to each other, separated from the W-C- cheese cluster by the second axis.

**Fig 4:**
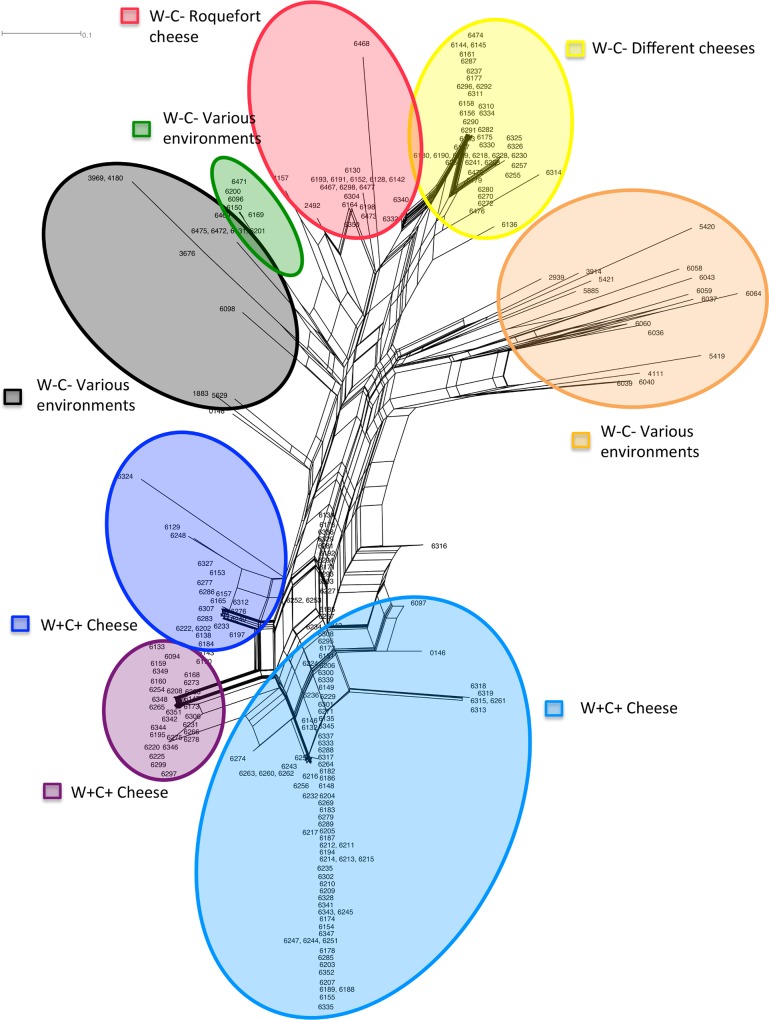
Splitstree of *Penicillium roqueforti* strains. Reticulation indicates likely occurrence of recombination. The colors correspond to those in Fig 1 of the clusters identified using Structure. The name of the clusters indicate whether they possess the *Wally* and *CheesyTer* genomic islands (W+C+) or not (W-C-) and whether they have been collected from cheeses, belonging or not to the Roquefort protected designation of origin (PDO), or from other environments, such as silage or spoiled food.

The Splitstree also discriminated the same eight genetic clusters and inferred similar genetic relationships among them as the DAPC (Fig 4). The Splitstree showed some reticulation, indicating recombination events, at least relatively recently.

## Discussion

Here, we used the strain collection from worldwide cheeses published recently [11], as well as the available public collection LCP MNHN, to reinvestigate the genetic structure of *P*. *roqueforti*. Overall, we detected in *P*. *roqueforti* a genetic structure consistent with previous studies [9–11], although with finer genetic subdivision. Indeed we found evidence for the existence of eight genetic clusters that segregated to some extent according to the environment of collection or to protected designation of origin (PDO). A cluster included only strains from other environments than cheeses and two clusters encompassed only strains from the Roquefort PDO. Despite the genetic structure at this level being subtle, the clear delimitation using STRUCTURE and the significant *F*_*ST*_ showed that the differentiation was genuine. The additional population structure revealed here may be relevant for cheese-makers, as phenotypic differences have been reported between the previously identified clusters in *P*. *roqueforti* [9]. Specific metabolic or morphologic traits could indeed be looked for in the additional clusters revealed here, that could impact cheese characteristics. In particular, the genetic cluster encompassing only Roquefort strains may display specific traits. Genomic studies could then investigate how these traits have evolved.

The finer population structure revealed here compared to previous studies may also allow better understanding the history of domestication in *P*. *roqueforti*. Indeed, the DAPC and the Splitstree both show that the W+C+ and W-C- cheese clusters are not the most closely related, which could not be seen in previous studies considering only the three main genetic clusters. These relationships suggest two different independent origins of the strains used in cheese production. These two cheese main groups correspond to the W+C+ and W-C- cheese clusters, respectively, and harbour different growth rates [13] and different fertility levels [11]. This may be due to independent selection of strains with different traits for making specific cheeses or selection post-isolation.

Interestingly, the yellow W-C- cheese cluster harbored a few strains carrying the *Wallaby* and *CheesyTer*. The strains did not appear intermediated between the W+C+ and W-C- strains in any analysis, rendering the hypothesis of hybridization unlikely. These strains may instead have acquired the two genomic islands recently by horizontal gene transfers. The mechanisms involved are still unknown but horizontal gene transfers among *Penicillium* strains seem frequent [13]. They could be facilitated by the co-occurrence of W+C+ and W-C- cheese strains in the same individual cheeses [13].

Our analyses exploring finer genetic subdivision within *P*. *roqueforti* than previous studies thus bring new, interesting results, improving our understanding of the history of domestication. It should be noted, however, that the environments other than cheeses from where strains could be collected were mostly anthropogenic, i.e., silage and spoiled food, and may not correspond to the exact wild population of origin. Strains collected as food spoiler could even correspond to feral individuals. This may be the case in particular for the few strains clustering within cheese clusters. However, the finding that several well-delimited genetic clusters correspond to non-cheese strains, together with the fact that they include strains from wood, suggest that most of the strains from spoiled food and silage represent genetically isolated populations, and not only feral strains. Indeed, if food spoiler strains were feral cheese strains, they would cluster within our “cheese clusters”, which was not the case.

## Supporting information

S1 Table Strain ID and information: environment of collection and assignment to the eight clusters. (XLSX)
